# The ultimate and proximate mechanisms driving the evolution of long tails in forest deer mice

**DOI:** 10.1111/evo.13150

**Published:** 2016-12-27

**Authors:** Evan P. Kingsley, Krzysztof M. Kozak, Susanne P. Pfeifer, Dou‐Shuan Yang, Hopi E. Hoekstra

**Affiliations:** ^1^ Howard Hughes Medical Institute, Department of Organismic and Evolutionary Biology, Department of Molecular and Cellular Biology, Museum of Comparative ZoologyHarvard UniversityCambridgeMassachusetts02138; ^2^ Department of ZoologyUniversity of CambridgeCambridgeCB2 3EJUnited Kingdom; ^3^ Current Address: Smithsonian Tropical Research InstituteApartado Postal 0843–03092PanamáRepública de Panamá; ^4^ School of Life Sciences, École Polytechnique Fédérale de Lausanne, Lausanne, Switzerland; Swiss Institute of Bioinformatics, Lausanne, Switzerland and School of Life SciencesArizona State UniversityTempeArizona85287; ^5^ Burke Museum and Department of BiologyUniversity of WashingtonSeattleWashington98195; ^6^ Current Address: US Fish and Wildlife ServiceVentura Field Office, 2493 Portola Road #BVenturaCalifornia93003

**Keywords:** Caudal vertebrae, convergence, local adaptation, parallel evolution, *Peromyscus maniculatus*, skeletal evolution

## Abstract

Understanding both the role of selection in driving phenotypic change and its underlying genetic basis remain major challenges in evolutionary biology. Here, we use modern tools to revisit a classic system of local adaptation in the North American deer mouse, *Peromyscus maniculatus*, which occupies two main habitat types: prairie and forest. Using historical collections, we find that forest‐dwelling mice have longer tails than those from nonforested habitat, even when we account for individual and population relatedness. Using genome‐wide SNP data, we show that mice from forested habitats in the eastern and western parts of their range form separate clades, suggesting that increased tail length evolved independently. We find that forest mice in the east and west have both more and longer caudal vertebrae, but not trunk vertebrae, than nearby prairie forms. By intercrossing prairie and forest mice, we show that the number and length of caudal vertebrae are not correlated in this recombinant population, indicating that variation in these traits is controlled by separate genetic loci. Together, these results demonstrate convergent evolution of the long‐tailed forest phenotype through two distinct genetic mechanisms, affecting number and length of vertebrae, and suggest that these morphological changes—either independently or together—are adaptive.

Understanding both the ultimate and proximate mechanisms driving adaptation remains a major challenge in biology. One way researchers have implicated a role of natural selection in driving phenotypic change is to show the repeated evolution of a trait in similar environments. Such correlations, when controlled for phylogenetic relatedness, can provide evidence for selection rather than stochastic processes driving the evolution of a trait of interest (Felsenstein 1985; Harvey and Pagel 1991). Because trait evolution depends on genetic change, we can gain a deeper understanding of adaptation by also studying its underlying genetic basis. Understanding the genetic architecture of convergent evolution can inform us about the roles of selection and constraint and how these processes may affect evolutionary outcomes (Arendt and Reznick 2008; Manceau et al. 2010; Elmer and Meyer 2011; Losos 2011; Martin and Orgogozo 2013; Rosenblum et al. 2014). Thus, by studying multiple levels of evolution–‐from organisms to genomes–‐we have a much more complete picture of the adaptive process.

Variation among populations of the deer mouse, *Peromyscus maniculatus*, provides a system for understanding both the organismal and genetic basis of evolution by local adaptation. This species has the widest range of any North American mammal (Hall 1981), and populations are adapted to their local environments in many parts of the range (Fig. 1A; e.g., Dice 1947; Hammond et al. 1999; Storz et al. 2007; Linnen et al. 2009; Bedford and Hoekstra 2015). Perhaps most strikingly, following the Pleistocene glacial maximum in North America, mice migrated from southern grassland habitat northward and colonized forested habitats, where they have become more arboreal (Hibbard 1968). Natural historians have long recognized two ecotypes of deer mice: forest‐dwelling and prairie‐dwelling forms (e.g., Dice 1940; Hooper 1942; Blair 1950). These ecotypes are differentiated both behaviorally and morphologically; mice found in forests tend to have smaller home ranges (Blair 1942; Howard 1949), prefer forest habitat over prairie habitat (Harris 1952), and prefer elevated perches (Horner 1954) as well as have bigger ears, longer hind feet, and longer tails (Osgood 1909; Blair 1950; Horner 1954) than prairie forms. However, it is unknown if the current, widespread forest populations reflect a single origin of the arboreal morphology that has spread across the continent, or if independent lineages have converged on the forest phenotype, and the genetic architecture underlying these phenotypic changes remains unexamined.

**Figure 1:**
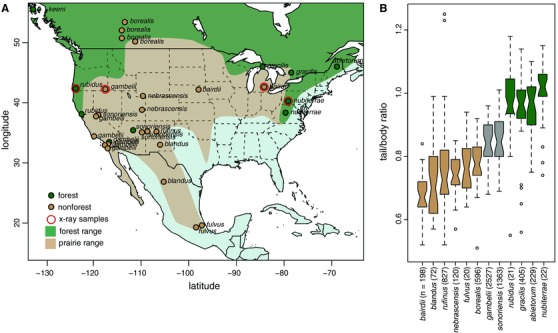
Deer mouse geography and tail length variation. (A) Map of North America showing the roughly defined range of *P. maniculatus*. Broad‐scale forest (green) and prairie (tan) range limits are shown, following Osgood (1909) and Hall (1981). Each dot represents a collecting locale from which one to nine samples were obtained. Dot color represents the local GIS land cover‐defined habitat of the site (green = forest, tan = nonforest). Mice from all sample locales were used in subsequent genome‐wide capture analyses. The red outline for “*x*‐ray samples” indicates that we used samples from those locations for the comparison of vertebral number and length. (B) Box‐and‐whisker plot of tail/body length ratio variation among deer mouse subspecies from museum collections. Box color indicates local habitat in which samples were collected based on GIS land cover data for those subspecies; green = forest, tan = nonforest, gray = mixed. (“Mixed” indicates that populations from a single subspecies were captured in both forest and nonforest locations.) [Color figure can be viewed at wileyonlinelibrary.com]

Of the morphological traits associated with forest habitats, arguably the best recognized is tail length, and evidence suggests that tail elongation may be an adaptive response to increased arboreality. Previous work has shown that deer mice use their tails extensively while climbing. For example, Horner (1954) carried out elegant experiments on climbing behavior in *Peromyscus*, and not only found a correlation between tail length and climbing performance, but also provided experimental evidence that within *P. maniculatus*, forest mice are more proficient climbers than their short‐tailed counterparts and that they rely on their tails for this ability. Moreover, in two other *Peromyscus* species, tail length correlates with degree of arboreality (Smartt and Lemen 1980), and climbing ability has been shown to be heritable (Thompson 1990).

Here, we investigate the evolution of the deer mouse tail in several complementary ways. First, we reconstruct phylogeographic relationships among 31 populations of *P. maniculatus* to test hypotheses about the evolution of tail length. We show that forest‐dwelling deer mice do not belong to a single phyletic group or genetic cluster, and thus that long‐tailed forest forms appear to have independently converged. We also demonstrate that the evolution of longer tails is correlated with living in forest habitats, even when taking account of relatedness among populations. Second, we investigate the morphological basis of tail length differences in two geographically distant populations, implicating similar mechanisms of tail elongation in eastern and western forest populations. Finally, we show that differences in the constituent traits of tail length between forest and prairie mice, despite correlation of these traits in the wild, are genetically separable in recombinant laboratory populations. Together, these results suggest that natural selection maintains multiple independent locally adapted forest populations and that independent genetic loci contribute to increases in vertebral number and length.

## Methods

### SAMPLES OF MUSEUM SPECIMENS

To quantify the degree of variation in overall tail length in this widespread species, we downloaded records of *P. maniculatus* from the Mammal Network Information System (MaNIS 2015), the Arctos database (Arctos 2015), and individual museum databases. Because nearly all specimens were present in the collection as prepared skins, we considered the original collector's field data to be the most reliable source of measurements. We excluded all specimens labeled as “juvenile,” “subadult,” or “young adult” or those having any tail abnormalities or injuries. We also removed any individuals with total length below 106 mm or tail length below 46 mm, which are considered the adult minima for this species (Hall 1981; Zheng et al. 2003). In total, we gathered data from 6400 specimens.

To assign specimens to subspecies, we digitally scanned the original distribution maps from Hall (1981) and georeferenced them in ArcGIS v. 9.2 (ESRI). *P. maniculatus* has highly stable subspecies ranges and most of the named subspecies have been described as forest or prairie types based on morphology and inspection of habitat (King 1968; Hall 1981; Gunn and Greenbaum 1986). We found that these historical assignments in the majority of cases hold true: based on GIS land cover data, we could assign most of the specimens labeled as a particular subspecies to a single habitat type. Two subspecies, *P. m. gambelii* and *P. m. sonoriensis*, were captured in both forest and nonforest habitats, so we have called their habitat type “mixed.”

### MORPHOMETRIC MEASUREMENTS AND STATISTICS

We calculated two types of dependent variables. First, we calculated the ratio of tail length to body length for all individuals. Second, we addressed potential nonlinear scaling of the two measures by fitting a linear model of tail length versus body length (Fox and Weisberg 2011). Models, including log, square, and Box‐Cox transformations of the response variable, as well as quadratic and cubic terms for the predictor, were compared using ANOVA and the adjusted *R* ^2^ values. Because the best model had very low explanatory power (*R* ^2 ^ = 0.11), we decided that simple ratios are the best statistic. As the ratios were not normally distributed (*P* < 0.001, Kolmogorov–Smirnoff test), the means of prairie, forest, and unclassified forms were compared with the Kruskal–Wallis nonparametric ANOVA. The R package *car* was used for all computations (R Development Core Team 2005; Fox and Weisberg 2011).

### MORPHOLOGY AND HABITAT DESIGNATION

Because skeletal preparations of museum specimens often do not have complete tails (e.g., the smallest vertebrae are often lost during sample preparation), we conducted more detailed analyses on whole mouse specimens (i.e., frozen or fluid‐preserved). We focused the next set of genomic and morphometric analyses on 80 individuals from our lab collection as well as loans from researchers and museums, covering multiple regions included in the above analyses (see Table S1 for details). The sampling encompassed the range of *P. maniculatus* and included 31 locations, spread across multiple habitats (Fig. 1A).

We assigned the animals from both datasets to local habitat types based on their specific sampling locations: we used ArcGIS (ESRI) to extract land cover (i.e., habitat type) information from the North American Land Change Monitoring System 2010 Land Cover Database (NALCMS 2010) with a 1 km‐radius buffer around each sampling location. We split land cover categories into forest and nonforest/prairie designations for all analyses: we called classes 1–6 and 14 forest (Temperate or subpolar needleleaf forest, Subpolar taiga needleleaf forest, Tropical or subtropical broadleaf evergreen forest, Tropical or subtropical broadleaf deciduous forest, Temperate or subpolar broadleaf deciduous forest, Mixed Forest, Wetland) and others nonforest/prairie (Tropical or subtropical shrubland, Temperate, or subpolar shrubland, Tropical or subtropical grassland, Temperate or subpolar grassland, Cropland, Urban, and Built‐up).

### ARRAY‐BASED CAPTURE AND SEQUENCING OF SHORT‐READ LIBRARIES

To assess genome‐wide population structure, we used an array‐based capture library and sequenced region‐enriched genomic libraries using an Illumina platform (Gnirke et al. 2009). Our MYselect (MYcroarray; Ann Arbor, MI) capture library sequences include probes for 5114 regions of the *Peromyscus maniculatus* genome, each averaging 1.5 kb in length (totaling 5.2 Mb of unique, nonrepetitive sequence; see Domingues et al. [2012] and Linnen et al. [2013]). We extracted genomic DNA using DNeasy kits (Qiagen; Germantown, MD) or the Autogenprep 965 (Autogen; Holliston, MA) and quantified it using Quant‐it (Life Technologies). 1.5 μg of each sample was sonically sheared by Covaris (Woburn, MA) to an average size of 200 bp, and Illumina sequencing libraries were prepared and enriched following Domingues et al. (2012). Briefly, we prepared multiplexed sequencing libraries in five pools of 16 individuals each using a “with‐bead” protocol (Fisher et al. 2011) and enriched the libraries following the MYselect protocol. We pulled down enrichment targets with magnetic beads (Dynabeads, Life Technologies; Carlsbad, CA), PCR amplified with universal primers (Gnirke et al. 2009), and generated 150 bp paired end reads on a HiSeq2000 (Illumina Inc.; San Diego, CA).

### SEQUENCE ALIGNMENT

We preprocessed raw reads (fastq files) by removing any potential nontarget species sequence (e.g., from sequence adapters) and by trimming low quality ends using cutadapt v. 1.8 (Martin 2011) and Trim Galore! v. 0.3.7 (options: *‐q 20 –phred33 –stringency 3 –length 20 –paired –retain_unpaired*) before aligning them to the Pman_1.0 reference assembly using Stampy v. 1.0.22 (Lunter and Goodson 2011) with default parameters. We removed optical duplicates using SAMtools v. 1.2 (Li et al. 2009), retaining the read pair with the highest mapping quality. Prior to calling variants, we performed a multiple sequence alignment using the Genome Analysis Toolkit v. 3.3 (GATK) IndelRealigner tool (McKenna et al. 2010; DePristo et al. 2011; Van der Auwera et al. 2013). Following GATK's Best Practice recommendations, we computed Per‐Base Alignment Qualities (BAQ) (Li 2011), merged reads originating from a single sample across different lanes, and removed PCR duplicates using SAMtools v. 1.2. To obtain consistent alignments across all lanes within a sample, we conducted a second multiple sequence alignment and recalculated BAQ scores. We finally limited our dataset to proper pairs using SAMtools v. 1.2.

### VARIANT CALLING AND FILTERING

We used GATK's HaplotypeCaller to generate initial variant calls via local de novo assembly of haplotypes. We combined the resulting gvcf files using GATK's CombineGVCFs command and jointly genotyped the samples using GATK's GenotypeGVCFs tool. After genotyping, we filtered these initial variant calls using GATK's VariantFiltration to minimize the number of false positives in the dataset. In particular, we applied the following set of filter criteria: we excluded SNPs for which three or more variants were found within a 10 bp surrounding window (clusterWindowSize = 10) to eliminate redundant variants; there was evidence of a strand bias as estimated by Fisher's exact test (FS > 60.0); the read mapping quality was low (MQ < 60); or the mean of all genotype qualities was low (GQ_MEAN < 20). We limited the variant dataset to biallelic sites using VCFtools (Danecek et al. 2011) and excluded genomic positions that fell within repeat regions of the reference assembly (as determined by RepeatMasker [Smit et al. 2013–2015]). We excluded genotypes with a genotype quality score of less than 20 (corresponding to P[error] = 0.01) or a read depth of less than four using VCFtools to minimize genotyping errors. In addition, we filtered variants on the basis of Hardy Weinberg Equilibrium (HWE) to remove variants that might be influenced by selection and thus distort phylogenetic relationships: a *P*‐value for HWE was calculated for each variant using VCFtools, and variants with *P* < 0.01 were removed. This HWE filtering excluded <0.7% of sites.

### GENETIC PRINCIPAL COMPONENTS ANALYSIS (PCA)

To assess genetic structure across the range of *P. maniculatus*, we first used SMARTPCA and TWSTATS (Patterson et al. 2006) with the genome‐wide SNP data (after filtering for variants genotyped in >50% of individuals, the final dataset contained 7396 variants) from the enriched short‐read libraries described above. We detected significant structure in the first two principal components (*P* < 0.05 by Tracy–Widom theory, as applied in TWSTATS). To further visualize genetic relationships inferred by the PCA, we generated a neighbor‐joining tree by computing Euclidean distances between individuals in the significant eigenvectors of the SMARTPCA output. The Python code to produce these trees can be found at github.com/kingsleyevan/phylo_epk.

### PHYLOGEOGRAPHY AND MONOPHYLY OF FOREST FORMS

We filtered the capture data to include only the variable sites with information for at least four individuals (14,076 SNPs) and formatted the resultant data in PLINK v. 1.8 (Purcell et al. 2007). The VCF was converted to a fasta alignment using PGDSpider v. 2.0 (Lischer and Excoffier 2012). We generated a phylogenetic tree in RAxML v. 8 assuming the GTR+G model with 500 bootstrap replicates and a correction for ascertainment bias (Stamatakis 2014). We then tested the hypothesis that long‐tailed forest individuals are monophyletic, either across the continent or within each of the western and eastern clades, by constraining the tree inference and comparing the likelihood using the Approximately Unbiased test (AU) (Shimodaira 2002) in CONSEL v. 0.2 (Shimodaira and Hasegawa 2001).

### MITOCHONDRIAL DNA ANALYSIS

Mitochondrial sequences were generated for a set of 106 samples, including 35 of those used in the capture experiment as well as *P. keeni, P. polionotus*, and *P. leucopus* as outgroups (Table S2). Amplification and sequencing were performed following Hoekstra et al. (2005). Briefly, we amplified the region spanning the single‐exon gene CO3 and ND3, with the intervening Glycine tRNA, in a single PCR, and Sanger‐sequenced using the primers 5′ CATAATCTAATGAGTCGAAATC 3′ (forward) and 5′ GCWGTMGCMWTWATYCAWGC 3′ (reverse). We aligned the resultant 1190 bp sequences using MUSCLE (Edgar 2004). The ML tree estimation and monophyly testing were carried out as above, with a separate partition defined for each codon position.

### WITHIN‐SPECIES COMPARATIVE ANALYSIS

To test for a correlation between tail length and habitat type, we used a comparative approach that accounts for the phylogenetic relationships among samples. First, we calculated standard Phylogenetically Independent Contrasts (Felsenstein 1985), based on both the nuclear and mitochondrial ML phylogenies, using the R package GEIGER (Harmon et al. 2008). Because we are comparing populations within a single species, this analysis is potentially confounded by nonindependence because of gene flow in addition to shared ancestry (Stone et al. 2011). Nonetheless, these results support the conclusions of the two controlled tests described below.

To control for the nonindependence of populations outside the context of bifurcating tree, we used a generalized linear‐mixed model approach as described by Stone et al. (2011). We used two measures of genetic similarity. First, we pared our data down to populations from which we had three or more individuals (now six populations) and estimated global mean and weighted F_ST_ between populations using the method of Weir and Cockerham (1984) as implemented in VCFtools (Danecek et al. 2011; see “in population contrasts” in Table S1). Second, we used a measure of genetic similarity (“–relatedness2” in VCFtools: the kinship coefficient of Manichaikul et al. 2010) between all individuals in the dataset for which we have tail and body measurements (“in individual contrasts” in Table S1). For both the population‐ and individual‐level analyses, we used a generalized linear mixed model approach, as implemented in the R package *MCMCglmm* (Hadfield 2010) to test for an effect of habitat (forest vs nonforest) on population average tail/body ratio or individual tail/body ratio when including F_ST_ or kinship coefficient as a random effect.

### VERTEBRAL MORPHOMETRICS OF WILD‐CAUGHT SPECIMENS

To measure tail morphology, we *x*‐ray imaged the skeletons of wild‐caught mice from four populations (Fig. 1A [red circles], and see Table S1 for specimen details). We used a Varian *x*‐ray source and digital imaging panel in the Museum of Comparative Zoology Digital Imaging Facility. To measure the lengths of individual vertebra we used ImageJ's segmented line tool, starting from the first sacral vertebra and proceeding posterior to the end of the tail. The boundary between the sacral and caudal segments of the vertebral column is not always clear, so for consistency, we called the first six vertebrae (starting with the first sacral attached) the sacral vertebrae; the caudal vertebrae are all the vertebrae posterior to the sixth sacral vertebra. This approach allowed us to reliably compare vertebrae across individuals.

Because tail length scales with body size in our sample, we fitted a linear model with the *lm* function in R (R Development Core Team 2005) to adjust all vertebral length measurements for body size. We regressed total tail length (*R* ^2^ = 0.62) and the lengths of individual vertebrae (*R* ^2^ = 0.14–0.59) on the sum length of the six sacral vertebrae and used the residuals from the linear fit for all subsequent analyses. We obtained similar results when we regressed vertebral length measurements on femur length instead of sacral vertebral length.

### F2 INTERCROSS TRAIT CORRELATION ANALYSIS

To examine the genetic architecture of tail traits, we conducted a genetic cross between a prairie and forest population. We originally obtained prairie deer mice, *P. m. bairdii*, from the Peromyscus Genetic Stock Center (University of South Carolina). We captured forest deer mice, *P. m. nubiterrae*, at the Powdermill Nature Reserve in Westmoreland County, Pennsylvania, and used 18 individuals to found a laboratory colony, after quarantine at Charles River Laboratories. All mice were housed in the Hoekstra lab at Harvard University. We mated a single male and female of each subspecies—two mating pairs, one in each cross direction—and used their offspring to establish 5 F1 sibling mating pairs. We then *x*‐ray imaged the resulting 96 F2 offspring and measured their tail morphology as described above (see section on Vertebral morphometrics). We used the *lm* function in R to assess correlations in the resulting measurements. All animals were adults between 80 and 100 days old when *x*‐rayed.

## Results

### FOREST DEER MICE DO NOT FORM A SINGLE CLADE

**Figure 2:**
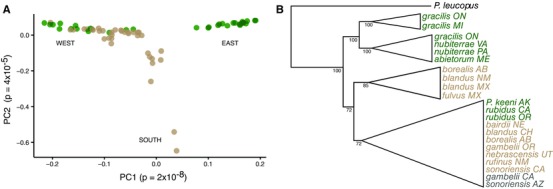
Deer mouse population structure and gene flow. (A) Plot of the first two principal components calculated from a genome‐wide sample of 7396 high‐quality SNPs from populations ranging across the continent (sampling locales shown in Fig. 1A). Each dot (*n* = 80) represents a single individual. (B) Cladogram based on nuclear Maximum Likelihood phylogeny collapsed to high‐confidence clades. Node labels represent bootstrap support. In both panels, colors indicate local GIS land cover‐defined habitat (tan = prairie, green = forest, gray = mixed). [Color figure can be viewed at wileyonlinelibrary.com]

We examined museum records of 6400 specimens belonging to 12 subspecies of *Peromyscus maniculatus* from across North America. Nearly all of the forest forms have substantially longer tails than the prairie populations (Kruskal–Wallis test, *P* = 0.001), often equaling the length of their body (Fig. 1B). We used an array‐based capture approach to resequence and call SNPs in >5000 genomic regions in a continent‐wide sample of animals from 31 *P. maniculatus* populations, *P. keeni*, and an outgroup *P. leucopus* (Fig. 1A). We identified 14,076 high‐quality SNPs, corresponding to an average of one variant every 187 kb of the reference genome with 24.73% of the genotypes missing. We explored genetic similarity among the sampled individuals by genetic principal components analysis (PCA; Patterson et al. 2006) and Maximum Likelihood phylogenetic inference (Fig. 2).

Both the PCA and phylogenetic methods show that individuals from forests (as determined by GIS) do not compose a single, monophyletic group. Instead, we see the mice of the putatively derived forest forms clustering with nearby nonforest forms in the PCA (Fig. 2A). Trees based on PCA distances (Fig. S1), estimated from DNA sequences (both genome‐wide SNPs [Fig. 2B; Fig. S2] and mtDNA [Fig. S3]), all identify multiple origins of forest forms. Monophyly is rejected in ML tests using the nuclear and mitochondrial data (AU test, *P* < 0.001). These results show that forest forms are evolving independently in eastern and western parts of the species range.

### VARIATION IN TAIL LENGTH CORRELATES WITH HABITAT

To assess whether differences in the length of the tail are significant even when accounting for evolutionary relationships among populations (created by gene flow and/or shared ancestry), we included measures of genetic similarity among populations and individuals in a set of generalized linear mixed models. In these models, we ask whether animals in different habitats have significantly different tail/body ratios when including measures of genetic similarity as random effects.

**Figure 3:**
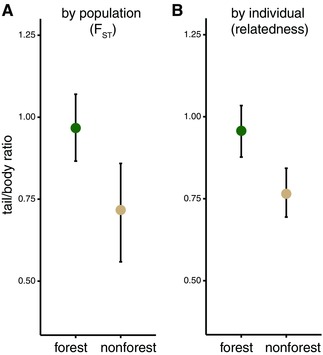
Tail length differences between habitats when accounting for genetic non‐independence. (A) Tail/body length ratios for each population predicted by a generalized linear‐mixed model taking genetic differentiation (F_ST_) between populations into account. (B) Tail/body length ratios for prairie and forest individuals predicted by a similar model as in A, but with pairwise genetic relatedness (kinship coefficient [Manichaikul et al. 2010]) between individuals taken into account. Error bars represent 95% confidence intervals. [Color figure can be viewed at wileyonlinelibrary.com]

First, we considered whether forest and prairie populations differ in their mean tail lengths. When taking pairwise F_ST_ between populations into account (Fig. S4), we find that habitat has a significant effect in our mixed model (*P* = 0.002 for weighted mean F_ST_, *P* < 0.001 for global mean F_ST_; fit by Markov Chain Monte Carlo, MCMC [Hadfield 2010]). Next, we assessed whether individuals from forest and prairie habitats differ in tail length when accounting for genetic similarity. We find a significant effect of habitat on tail/body ratio in our mixed model with kinship coefficient (Manichaikul et al. 2010) as a random effect (MCMC; *P* < 0.001). We show model‐predicted population means in Figure 3A and the predicted individual means by habitat in Figure 3B. In addition, phylogenetically independent contrasts show a correlation between habitat type and tail/body length ratio (adjusted *R* ^2^ = 0.30, *P* < 1 × 10^–4^). Together, these results robustly show that, even when taking nonindependence of populations into account, deer mice from forested habitats do indeed have significantly longer tails than those from prairie habitats.

### CONVERGENCE IN SKELETAL MORPHOLOGY

**Figure 4:**
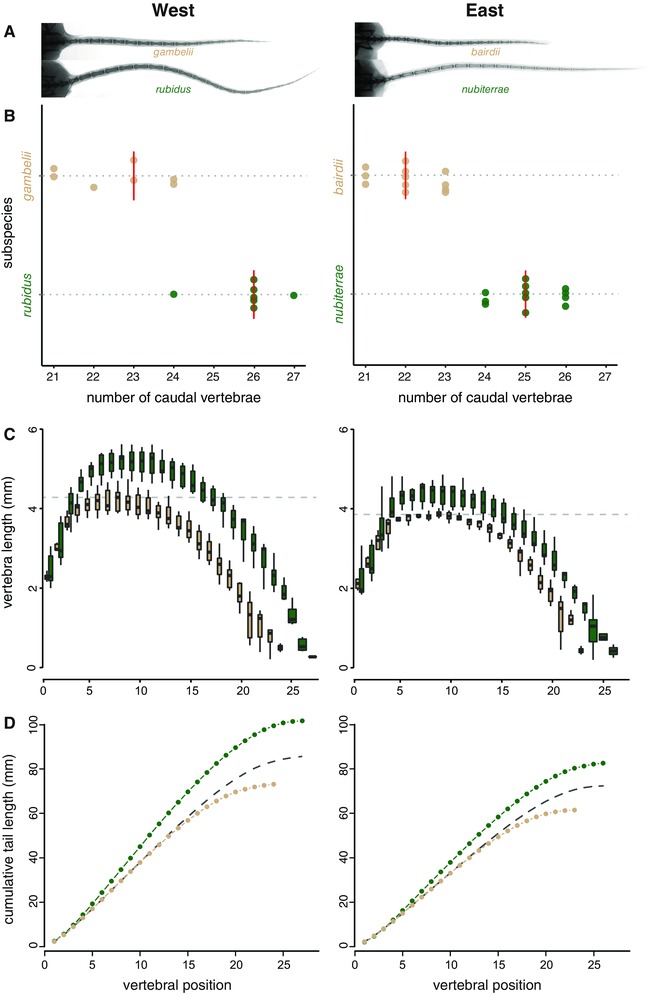
Convergent tail vertebral morphology in eastern and western forest‐prairie population pairs. (A) Representative radiographs of deer mouse tails from the eastern and western population pairs. (B) Forest mice have more caudal vertebrae than prairie mice in the east and west. Vertical red lines represent medians. Note truncated axis. (C) Forest mice have longer vertebrae than prairie mice in the east and west. Dashed line represents the median length of the longest prairie vertebra; the segment of the forest tail with longer vertebrae is 4–16 and 4–15 for western and eastern populations, respectively. (D) Summary of tail vertebral differences between forest and prairie mice. Dots represent mean cumulative tail lengths. Dashed line is the cumulative length of the mean prairie tail with three extra vertebrae added, which represents an estimate of the maximum contribution of difference in number of vertebrae to the difference in total length. For C and D, “vertebral position” indicates the *n*th caudal vertebra, starting from the base of the tail. [Color figure can be viewed at wileyonlinelibrary.com]

We *x*‐rayed specimens from two pairs of geographically distant forest‐prairie populations (Fig. 1A [red circles] and “*x*‐ray samples” in Table S1) and measured their vertebrae. We focused on these four populations because individuals from the two forest populations represent independently evolving forest lineages. Though both forest populations have longer tails than nonforest populations, eastern and western forest mice do not have identical tail lengths: western mice have longer tails, both absolutely and relative to their body size, than eastern forest mice (Fig. 4A–D).

In addition to the total tail length, we measured the number of caudal vertebrae and the lengths of each caudal vertebra. We found that, in both east and west paired comparisons, forest mice had significantly longer tails, significantly more vertebrae, and significantly longer vertebrae (here we tested the single longest vertebra) than their nearby prairie form (Fig. 4; Wilcoxon tests, *P* < 1.6 × 10^–4^ for all comparisons). Notably, we performed all these tests on body‐size‐corrected data, which means that these forest‐prairie differences are not driven by overall differences in body length. We also found significant effects of habitat and subspecies on tail length and longest vertebra length in a two‐way analysis of covariance (ANCOVA) on log‐transformed data with sacral length as a covariate (*P* < 6.6 × 10^–8^ for all effects).

First, we counted the number of caudal vertebrae and found that forest forms have significantly more vertebrae than the prairie forms (Kruskal–Wallis test, *Χ* ^2^ = 29.0, *P* = 7.2 × 10^–8^). In both cases, we found that forest mice had, on average, three more vertebrae than the nearby prairie form. However, the western mice had, on average, one more caudal vertebra than the eastern population from the same habitat type (Fig. 4B). Importantly, none of the populations can be distinguished by the number of trunk vertebrae (all samples had 18 or 19), showing that vertebral differences are specific to the tail.

We next compared the lengths of the individual caudal vertebrae along the tail and found that many but not all caudal vertebrae are longer in the forest than in prairie mice. In the eastern population pair, caudal vertebrae 4 through 15 had longer median lengths in the forest mice than the longest caudal vertebra in prairie mice. The corresponding segment in the western population pair is caudal vertebrae 4 through 16 (Fig. 4C).

We also estimated the relative contributions of differences in vertebral length and vertebral number to the overall difference in tail length. To do this, we modeled the forest and prairie mice having an equal number of vertebrae by inserting three long vertebrae into the center of the prairie tails. These simulated “prairie+3” tails compensated for 42% and 53% of the average difference in overall tail length between the eastern and western forms, respectively (Fig. 4D). These estimates represent an upper bound of the contribution of the difference in vertebral number relative to vertebral length in these populations. Finally, a linear model of the form *total length* ∼ *longest vertebra length + number of caudal vertebrae* has an *R* ^2^ = 0.98, suggesting that differences in length and number of vertebrae explain nearly all of the difference in total tail length between these populations. Thus, these two morphological traits—length and number of vertebrae—contribute approximately equally to the difference in overall tail length in the western and eastern clades.

### VERTEBRAE LENGTH AND VERTEBRAE NUMBER ARE GENETICALLY SEPARABLE

**Figure 5:**
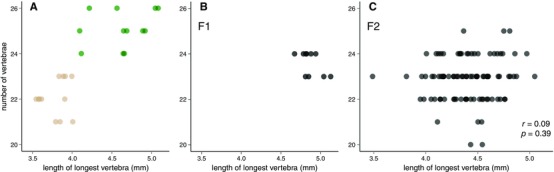
No significant correlation between number and length of caudal vertebrae in a laboratory intercross. Each point represents the number of caudal vertebrae and the length of the longest caudal vertebra measured from a radiograph of a parental type (A, *P. m. nubiterrae* (green), *P. m. bairdii* (tan), *n* = 12 of each subspecies), or first‐generation F1 (B, *n* = 10) or second‐generation F2 (C, *n* = 96) *nubiterrae* x *bairdii* hybrids. Ninety‐six F2 individuals allow 80% power to detect a correlation of *r* > 0.25. [Color figure can be viewed at wileyonlinelibrary.com]

Among the four populations examined above, the two forest populations have both more caudal vertebrae and longer caudal vertebrae. To test whether these two morphological differences are controlled by the same or distinct genetic loci, we performed a reciprocal intercross between the eastern populations: long‐tailed, forest *P. m. nubiterrae*, and short‐tailed, prairie *P. m. bairdii* (Fig. 5A). Specifically, we generated 10 F1 hybrids and paired those 10 F1s to produce 96 F2 recombinant individuals. Using vertebra measurements from *x*‐ray images of each of these animals, we show that alleles controlling number of vertebrae act in a semidominant manner as evidenced by intermediate phenotypes in first generation (F1) hybrids, whereas length of vertebrae in F1 hybrids is more similar to the forest form, suggesting the *P. m. nubiterrae* allele(s) are primarily dominant (Fig. 5B). Next, we tested for a correlation between the number of caudal vertebrae and the length of the longest caudal vertebra. We detect no significant correlation (Fig. 5C; *t* = 0.87, df = 94, *P* = 0.39) between vertebral length and vertebral number in the tails of our F2 animals. A sample of 96 individuals is well powered, allowing an 80% probability of detecting a correlation of *r* > 0.25 at *P* < 0.05. These data suggest that differences in these two phenotypic traits—vertebral number and length—are under independent genetic control.

## Discussion

In this study, we explored the evolution of a repeated phenotype within a single species. We found that long‐tailed forest‐dwelling deer mice are evolving independently in eastern and western parts of their range and that tail length does indeed differ between forest and nonforest habitats, even when controlling for genetic relatedness. Furthermore, we showed that longer tails, in both eastern and western forest mice, are a result of differences in both the number of caudal vertebrae and also the lengths of those vertebrae. Finally, we demonstrated that, despite the observation that caudal vertebrae number and length are correlated in nature, the genetic mechanisms producing these differences can be decoupled in a laboratory intercross. Together, these results imply that natural selection is driving differences in tail length and provide insight into the genetic architecture that underlies tail length evolution.

In his large‐scale 1950 survey of adaptive variation in *Peromyscus*, W. Frank Blair described two main forms of *P. maniculatus*, grassland and forest, supporting previous suggestions (Osgood 1909). Therefore, we examined the phylogeography of the deer mouse in the context of intraspecific ecological adaptation, and specifically in the framework of the classic prairie‐forest dichotomy that has been recognized in this species for over one hundred years. Previous work on the phylogenetic relationships among deer mouse subspecies has hypothesized convergence in these forms—allozyme (Avise et al. 1979) and mitochondrial studies (Lansman et al. 1983; Dragoo et al. 2006) hinted at a split between eastern and western forest populations—yet none have explicitly considered morphological and ecological context in a continent‐wide sampling. Additionally, only the mitochondrial study of Dragoo et al. (2006) investigated population differentiation at a fine scale, but mitochondrial‐nuclear discordance in this species (e.g., Yang and Kenagy 2009; Taylor and Hoffman 2012) complicates the interpretation. Here, we used > 7000 genome‐wide SNPs to directly test for a correlation between habitat and morphology. We chose to use both tree‐based and non‐tree‐based methods, given the difficulty of constructing bifurcating phylogenetic relationships among intraspecific samples from populations experiencing gene flow.

The results of our genetic PCA provide strong evidence that forest mice do not form a single genetic cluster. The pattern of genomic differentiation we see in the PCA is roughly similar to that recovered by Avise et al. (1979), with a group of populations from eastern North America that are clearly separated from populations in the western half of the continent. In addition, we find a similar pattern and extent of divergence in Maximum Likelihood phylogenetic reconstruction among individuals. These results show at least two, one eastern and one western, independently evolving groups of forest *P. maniculatus*.

When we assigned habitat values to those populations using GIS land cover data, we found that populations captured in forest habitats have longer tails than those in nonforest habitats, even when taking genetic relatedness among populations into account. This is a critical finding that suggests that tail length differences are driven by natural selection: despite a century of investigation, the current study provides the first rigorous evidence for a continent‐wide correlation between tail length and habitat type in this species. Thus, we support the conjecture of Lansman et al. (1983): “It is thus very probable that the currently recognized forest‐grassland division of *P. maniculatus* does not reflect a fundamental phylogenetic split. Rather, it is more likely that environmental selection pressures have led to the independent evolutionary appearance of these two morphs in different *maniculatus* lineages.” Our results showing the correlation of morphology with habitat and the presence of this correlation in independent populations imply convergence on the population level: similar environments resulting in similar morphologies in distinct lineages.

Several lines of evidence suggest that these independent extant forest forms may derive from a short‐tailed prairie‐dwelling ancestor. First, although our SNP‐based phylogeny does not imply an obvious ancestral state, the topology of our mtDNA phylogeny (Fig. S3) is consistent with a prairie ancestor to the *P. maniculatus* group. Second, three studies identified *P. melanotis*, found primarily in grassy plains and characterized by a very short tail (Álvarez‐Castañeda 2005), as the outgroup to the *P. maniculatus* species group (Bradley et al. 2007; Gering et al. 2009; Platt et al. 2015). Other close relatives of *P. maniculatus* are also relatively short‐tailed, with the exception of another climbing specialist, the cactus mouse *P. eremicus*. Third, several fossils attributed to *P. maniculatus* and dated to the Pleistocene late‐glacial period—during which the *P. maniculatus* group is thought to have radiated—were found in regions likely to be mainly scrub grasslands (Hibbard 1968; Bryant and Holloway 1985). While it is always challenging to infer ancestral phenotypic states, these data are consistent with the ancestral *P. maniculatus* being a short‐tailed grassland form from which at least two long‐tailed forest forms evolved.

How would the interpretation of our results change if the ancestral *P. maniculatus* were not short‐tailed prairie dwellers? One possibility is that the ancestral state was long‐tailed, implying the subsequent evolution of shorter‐tailed prairie populations in the middle of the continent and the retention of the ancestral state in the east and west. In that case, we could not rule out a single transition in tail length, from longer to shorter. A second possibility is that the ancestral population had an intermediate or polymorphic length tail, suggesting that there were also transitions to short tails that we did not address here. Regardless of the ancestral phenotype—short, long, intermediate, or polymorphic—we show that the eastern and western forest forms are convergently evolving at the population level. By this, we mean that similar environments (i.e., forests) appear to favor similar phenotypes (i.e., long tails) in two phylogenetic groups that are not closely related to each other (in terms of population differentiation within this species).

Furthermore, in two forest‐prairie population pairs, one eastern and one western, we find that both pairs differ in the two components of the caudal skeleton that could vary to produce differences in tail length, namely the number of tail vertebrae and the length of those vertebrae. This coupling of vertebral number and vertebral length could be explained in two ways: (1) number and length of vertebrae are controlled by identical, or linked, regions of the genome, or (2) multiple genetic variants controlling number and length of vertebrae have been independently selected in eastern and western populations. To distinguish between these hypotheses, we examined F2 hybrid individuals from a laboratory intercross between a forest form, *P. m. nubiterrae*, and a prairie form, *P. m. bairdii*. If differences in the number and length of vertebrae are controlled by variants in the same region(s) of the genome, we expect them to be correlated in the F2 individuals. On the other hand, if the two morphological traits are under control of variants in different genomic regions, recombination should decouple these traits in the F2 generation, and we should detect no correlation between these traits. We found the latter: we detected no significant correlation between number and length of vertebrae in the F2. This result implies that eastern forest environments have favored alleles affecting differences in number and length at a minimum of two distinct genetic loci (i.e., one or more mutations that increase vertebrae number and elsewhere in the genome one or more mutations that increase vertebrae length). It may be that alleles affecting number and length were present in the source population from which the forest mice evolved, or that new mutations contributed to these distinct morphologies, or both. By identifying the alleles that influence tail length variation, future work can determine whether these locally adaptive alleles were selected from standing variation or have arisen independently from distinct mutations in eastern and western clades, and whether these mutations are shared in the eastern and western populations.

While vertebrae number and length evolved in tandem in our wild *Peromyscus* populations, this is contrary to what we may have expected based on artificial selection experiments. Rutledge et al. (1974) performed a selection study in which the authors selected for increased body length and tail length in replicate mouse strains. The authors found that, in two replicate lines selected for increased tail length, one line had evolved a greater number of vertebrae and the other evolved longer vertebrae. That the number and lengths of vertebrae can be genetically uncoupled may not be surprising, given the timing of processes in development that affect these traits. The process of somitogenesis, which creates segments in the embryo that presage the formation of vertebrae, is completed in the *Mus* embryo by 13.5 days of development (Tam 1981), while the formation of long bones does not begin until later in embryogenesis and skeletal growth continues well into the early life of the animal (Theiler 1989). Nonetheless, the contrasting result between natural and artificial selection studies suggests that it may be advantageous, biomechanically, to have both more and longer caudal vertebrae in wild populations that climb in forested habitats.

Together these data convincingly show that longer tails have evolved repeatedly in similar forested habitat, implicating a role of natural selection. Despite separate genetic mechanisms for number and length of vertebrae, both traits contribute approximately equally to the increase in tail length in both the eastern and western forest populations. Future work will explore the biomechanical implications of caudal vertebrae morphology on function (e.g., climbing), and ultimately fitness, as well as the underlying genetic and developmental mechanisms driving the repeated evolution of this adaptive phenotype.

Associate Editor: M. Streisfeld

Handling Editor: P. Tiffin

## Supporting information


**Figure S1**. Neighbor‐joining trees based on Euclidean distances in principal component space.
**Figure S2**. Maximum Likelihood phylogeny of *P. maniculatus* individuals based on >14000 nuclear SNPs.
**Figure S3**. Maximum Likelihood phylogeny of *P. maniculatus* individuals based on the mitochondrial CO3‐ND3 fragment.
**Figure S4**. Pairwise weighted F_ST_ among population samples containing >3 individuals.

**Table S1**. Samples genotyped in the genome‐wide capture.

**Table S2**. Samples genotyped at the mitochondrion.

## References

[evo13150-bib-0001] Álvarez‐Castañeda, S. T. 2005 *Peromyscus melanotis* . Mammalian Species 764:1–4.

[evo13150-bib-0002] Arctos: Collaborative Collections Management Solution . 2015 Available at <http://arctosdb.org>[accessed March 1, 2010].

[evo13150-bib-0003] Arendt, J. , and D. Reznick . 2008 Convergence and parallelism reconsidered: what have we learned about the genetics of adaptation? Trends Ecol. Evol. 23:26–32. 18022278 10.1016/j.tree.2007.09.011

[evo13150-bib-0004] Avise, J. C. , M. H. Smith , and R. K. Selander . 1979 Biochemical polymorphism and systematics in the genus *Peromyscus*. VII. Geographic differentiation in members of the *truei* and *maniculatus* species groups. J. Mammal. 60:177–192.

[evo13150-bib-0005] Babraham Bioinformatics: Trim Galore! 0.3.7. Available at <http://www.bioinformatics.babraham.ac.uk/projects/trim_galore>

[evo13150-bib-0006] Bedford, N. L. , and H. E. Hoekstra . 2015 *Peromyscus* mice as a model for studying natural variation. eLIFE 4:e06813. 10.7554/eLife.06813 PMC4470249 26083802

[evo13150-bib-0007] Blair, W. F. 1942 Size of home range and notes on the life history of the woodland deer‐mouse and eastern chipmunk in northern Michigan. J. Mammal. 23:27–36.

[evo13150-bib-0008] Blair, W. F. 1950 Ecological factors in speciation of *Peromyscus* . Evolution 4:253–275.

[evo13150-bib-0009] Bradley, R. D. , N. D. Durish , D. S. Rogers , J. R. Miller , M. D. Engstrom , and C. W. Kilpatrick . 2007 Toward a molecular phylogeny for *Peromyscus*: evidence from mitochondrial cytochrome‐*b* sequences. J. Mammal. 88:1146–1159. 19924266 10.1644/06-MAMM-A-342R.1 PMC2778318

[evo13150-bib-0010] Bryant, V. M. , and R. G. Holloway . 1985 A late‐Quaternary paleoenvironmental record of Texas: an overview of the pollen evidence In: Bryant V. M. and Holloway R. G., eds. Pollen records of late‐Quaternary North American sediments. American Association of Stratigraphic Palynologists, Dallas, TX.

[evo13150-bib-0011] Danecek, P. , A. Auton , G. Abecasis , C. A. Albers , E. Banks , M. A. DePristo , R. E. Handsaker , G. Lunter , G. T. Marth , S. T. Sherry , et al. 2011 The variant call format and VCFtools. Bioinformatics 27:2156–2158. 21653522 10.1093/bioinformatics/btr330 PMC3137218

[evo13150-bib-0012] DePristo, M. , E. Banks , R. Poplin , K. Garimella , J. Maguire , C. Hartl , A. Philippakis , G. del Angel , M. A. Rivas , M. Hanna , et al. 2011 A framework for variation discovery and genotyping using next‐generation DNA sequencing data. Nat. Genet. 43:491–498. 21478889 10.1038/ng.806 PMC3083463

[evo13150-bib-0013] Dice, L. R. 1940 Ecologic and genetic variability within species of *Peromyscus* . Am. Nat. 74:212–221.

[evo13150-bib-0014] Dice, L. R. 1947 Effectiveness of selection by owls of deer‐mice (*Peromyscus maniculatus*) which contrast in color with their background. Univ. of Mich. Contrib. Lab. Vert. Biol. 34:1–20.

[evo13150-bib-0015] Domingues, V. S. , Y.‐P. Poh , B. K. Peterson , P. S. Pennings , J. D. Jensen , and H. E. Hoekstra . 2012 Evidence of adaptation from ancestral variation in young populations of beach mice. Evolution 66:3209–3223. 23025610 10.1111/j.1558-5646.2012.01669.x

[evo13150-bib-0016] Dragoo, J. , J. Lackey , K. Moore , E. Lessa , J. Cook , and T. Yates . 2006 Phylogeography of the deer mouse (*Peromyscus maniculatus*) provides a predictive framework for research on hantaviruses. J. Gen. Virol. 87:1997–2003. 16760402 10.1099/vir.0.81576-0

[evo13150-bib-0017] Edgar, R. C. 2004 MUSCLE: multiple sequence alignment with high accuracy and high throughput. Nuc. Ac. Res. 32:1792–1797. 10.1093/nar/gkh340 PMC390337 15034147

[evo13150-bib-0018] Elmer, K. , and A. Meyer . 2011 Adaptation in the age of ecological genomics: insights from parallelism and convergence. Trends Ecol. Evol. 26:298–306. 21459472 10.1016/j.tree.2011.02.008

[evo13150-bib-0019] Felsenstein, J. 1985 Phylogenies and the comparative method. Am. Nat. 125:1–15.

[evo13150-bib-0020] Fisher, S. , A. Barry , J. Abreu , B. Minie , J. Nolan , T. M. Delorey , G. Young , T. J. Fennell , A. Allen , L. Ambrogio , et al. 2011 A scalable, fully automated process for construction of sequence‐ready human exome targeted capture libraries. Genome Biol. 12:R1. 21205303 10.1186/gb-2011-12-1-r1 PMC3091298

[evo13150-bib-0021] Fox, J. , and S. Weisberg . 2011 An R companion to applied regression, 2nd edn Sage, Thousand Oaks, CA.

[evo13150-bib-0022] Gering, E. J. , J. C. Opazo , and J. F. Storz . 2009 Molecular evolution of cytochrome *b* in high‐ and low‐altitude deer mice (genus *Peromyscus*). Heredity 102:226–235. 19107138 10.1038/hdy.2008.124 PMC4409697

[evo13150-bib-0023] Gunn, S. J. , and I. F Greenbaum . 1986 Systematic implications of karyotypic and morphologic variation in mainland *Peromyscus* from the Pacific Northwest. J. Mammal. 67:294–304.

[evo13150-bib-0024] Gnirke, A. , A. Melnikov , J. Maguire , P. Rogov , E. M. LeProust , W. Brockman , T. Fennell , G. Giannoukos , S. Fisher , C. Russ , et al. 2009 Solution hybrid selection with ultra‐long oligonucleotides for massively parallel targeted sequencing. Nat. Biotechnol. 27:182–189. 19182786 10.1038/nbt.1523 PMC2663421

[evo13150-bib-0025] Hadfield, J. D. 2010 MCMC methods for multi‐response generalized linear mixed models: the MCMCglmm R package. J. Stat. Softw. 33:1–22.20808728

[evo13150-bib-0026] Hall, E. R. 1981 The mammals of North America. Wiley. New York, NY.

[evo13150-bib-0027] Hammond, K. A. , J. Roth , D. N. Danes , M. R. Dohm . 1999 Morphological and physiological responses to altitude in deer mice *Peromyscus maniculatus* . Physiol. Biochem. Zool. 72:613–622. 10521329 10.1086/316697

[evo13150-bib-0227] Harmon, L. J. , J. T. Weir , C. D. Brock , R. E. Glor , and W. Challenger . 2008 GEIGER: investigating evolutionary radiations. Biochem. 24:129–131. 10.1093/bioinformatics/btm538 18006550

[evo13150-bib-0028] Harris, V. 1952 An experimental study of habitat selection by prairie and forest races of the deer mouse, *Peromyscus maniculatus* . Contrib. Lab. Vertebr. Biol. Univ. Mich. 56:1–53.

[evo13150-bib-0029] Harvey, P. H. , and M. D. Pagel . 1991 The comparative method in evolutionary biology. Oxford Univ. Press, Oxford.

[evo13150-bib-0030] Hibbard, C. W. 1968 Palaeontology In: King J. A., ed. Biology of *Peromyscus* (Rodentia). American Society of Mammalogists, Stillwater, OK.

[evo13150-bib-0031] Hoekstra, H. E. , J. G. Krenz , and M. W. Nachman . 2005 Local adaptation in the rock pocket mouse (*Chaetodipus intermedius*): natural selection and phylogenetic history of populations. Heredity 94:217–228. 15523507 10.1038/sj.hdy.6800600

[evo13150-bib-0032] Hooper, E. T. 1942 An effect on the *Peromyscus maniculatus* Rassenkreis of land utilization in Michigan. J. Mammal. 23:193–196.

[evo13150-bib-0033] Horner, B. E. 1954 Arboreal adaptations of *Peromyscus* with special reference to use of the tail. Univ. of Mich. Contrib. Lab. Vert. Biol. 61:1–84.

[evo13150-bib-0034] Howard, W. E. 1949 Dispersal, amount of inbreeding, and longevity in a local population of prairie deermice on the George Reserve, Southern Michigan. Univ. of Mich. Contrib. Lab. Vert. Biol. 43:1–50.

[evo13150-bib-0035] Lansman, R. A. , J. C. Avise , C. F. Aquadro , J. F. Shapira , and S. W. Daniel . 1983 Extensive genetic variation in mitochondrial DNA's among geographic populations of the deer mouse, *Peromyscus maniculatus* . Evolution 37:1–16. 10.1111/j.1558-5646.1983.tb05509.x 28568029

[evo13150-bib-0036] Li, H. 2009 The sequence alignment/map format and SAM tools. Bioinformatics 25:2078–2079. 19505943 10.1093/bioinformatics/btp352 PMC2723002

[evo13150-bib-0037] Li, H. 2011 Improving SNP discovery by base alignment quality. Bioinformatics 27:1157–1158. 21320865 10.1093/bioinformatics/btr076 PMC3072548

[evo13150-bib-0038] Linnen, C. R. , E. P. Kingsley , J. D. Jensen , and H. E. Hoekstra . 2009 On the origin and spread of an adaptive allele in deer mice. Science 325:1095–1098. 19713521 10.1126/science.1175826 PMC2736094

[evo13150-bib-0039] Linnen, C. R. , Y‐P. Poh , B. K. Peterson , R. D. Barrett , J. G. Larson , J. D. Jensen , and H. E. Hoekstra . 2013 Adaptive evolution of multiple traits through multiple mutations at a single gene. Science 339:1312–1316. 23493712 10.1126/science.1233213 PMC3836219

[evo13150-bib-0040] Lischer, H. E. L. , and L. Excoffier . 2011 PGDSpider: an automated data conversion tool for connecting population genetics and genomics programs. Bioinformatics 28:298–299. 22110245 10.1093/bioinformatics/btr642

[evo13150-bib-0041] Losos, J. B. 2011 Convergence, adaptation, and constraint. Evolution 65:1827–1840. 21729041 10.1111/j.1558-5646.2011.01289.x

[evo13150-bib-0042] Lunter, G. , and M. Goodson . 2011 Stampy: a statistical algorithm for sensitive and fast mapping of Illumina sequence reads. Genome Res. 21:936–939. 20980556 10.1101/gr.111120.110 PMC3106326

[evo13150-bib-0043] Manceau, M. , V. S. Domingues , C. R. Linnen , E. B. Rosenblum , and H. E. Hoekstra . 2010 Convergence in pigmentation at multiple levels: mutations, genes and function. Phil. Trans. R Soc. Lond. B Biol. Sci. 365:2439–2450. 20643733 10.1098/rstb.2010.0104 PMC2935106

[evo13150-bib-0044] Manichaikul, A. , J. Mychaleckyj , S. Rich , K. Daly , M. Sale , and W.‐M. Chen . 2010 Robust relationship inference in genome‐wide association studies. Bioinformatics 26:2867–2873. 20926424 10.1093/bioinformatics/btq559 PMC3025716

[evo13150-bib-0045] MaNIS: Mammal Networked Information System . 2015 Available at <http://manisnet.org>[Accessed 1 Mar. 2010].

[evo13150-bib-0046] Martin, M. 2011 Cutadapt removes adapter sequences from high‐throughput sequencing reads. EMBnet 17:10–12.

[evo13150-bib-0047] Martin, A. , and V. Orgogozo . 2013 The loci of repeated evolution: a catalog of genetic hotspots of phenotypic variation. Evolution 67:1235–1250. 23617905 10.1111/evo.12081

[evo13150-bib-0048] McKenna, A. , M. Hanna , E. Banks , A. Sivachenko , K. Cibulskis , A. Kernytsky , K. Garimella , D. Altschuler , S. Gabriel , M. Daly , et al. 2010 The Genome Analysis Toolkit: a MapReduce framework for analyzing next‐generation DNA sequencing data. Genome Res. 20:1297–1303. 20644199 10.1101/gr.107524.110 PMC2928508

[evo13150-bib-0049] Osgood, W. H. 1909 Revision of the Genus *Peromyscus*. North American Fauna. Washington, DC.

[evo13150-bib-0050] Patterson, N. , A. L. Price , and D. Reich . 2006 Population structure and eigenanalysis. PLoS Genet. 2:e190. 17194218 10.1371/journal.pgen.0020190 PMC1713260

[evo13150-bib-0051] Platt II, R. N. , C. W. Thompson , B. R. Amman , M. S. Corley , and R. D. Bradley . 2015 What is *Peromyscus*? Evidence from nuclear and mitochondrial DNA sequences for a new classification. J. Mammal. 96:708–719. 26937047 10.1093/jmammal/gyv067 PMC4668989

[evo13150-bib-0052] Purcell, S. , B. Neale , K. Todd‐Brown , L. Thomas , M. A. R. Ferreira , D. Bender , J. Maller , P. Sklar , P. I. W. de Bakker , M. J. Daly , et al. 2007 PLINK: a tool set for whole‐genome association and population‐based linkage analyses. Am. J. Hum. Gen. 81:559–575. 10.1086/519795 PMC1950838 17701901

[evo13150-bib-0053] Rosenblum, E. B. , C. E. Parent , and E. E. Brandt . 2014 The molecular basis of phenotypic convergence. Annu. Rev. Ecol. Evol. Syst. 45:203–226.

[evo13150-bib-0054] Rutledge, J. J. , E. J. Eisen , and J. E. Legates . 1974 Correlated response in skeletal traits and replicate variation in selected lines of mice. Theor. Appl. Genet. 45:26–31. 24419218 10.1007/BF00281170

[evo13150-bib-0055] Shimodaira, H. , and M. Hasegawa . 2001 CONSEL: for assessing the confidence of phylogenetic tree selection. Bioinformatics 17:1246–1247. 11751242 10.1093/bioinformatics/17.12.1246

[evo13150-bib-0056] Shimodaira, H. 2002 An approximately unbiased test of phylogenetic tree selection. Sys. Bio. 51:492–508. 10.1080/10635150290069913 12079646

[evo13150-bib-0057] Smartt, R. A. , and C. Lemen . 1980 Intrapopulational morphological variation as a predictor of feeding behavior in deermice. Am. Nat. 116:891–894.

[evo13150-bib-0058] Smit, A. F. A. , R. Hubley , and P. Green . RepeatMasker Open‐4.0 2013–2015. Available at <http://www.repeatmasker.org>

[evo13150-bib-0059] Snyder, L. 1981 Deer mouse hemoglobins: is there genetic adaptation to high altitude? Bioscience 31:299–304.

[evo13150-bib-0060] Stamatakis, A. 2014 RAxML Version 8: a tool for phylogenetic analysis and post‐analysis of large phylogenies. Bioinformatics 30:1312–1313. 24451623 10.1093/bioinformatics/btu033 PMC3998144

[evo13150-bib-0061] Stone, G. N. , S. Nee , and J. Felsenstein . 2011 Controlling for non‐independence in comparative analysis of patterns across populations within species. Philos. Trans. R. Soc. Lond. B Biol. Sci. 366:1410–1424. 21444315 10.1098/rstb.2010.0311 PMC3081573

[evo13150-bib-0062] Storz, J. F. , S. J. Sabatino , F. G. Hoffmann , and E. J. Gering . 2007 The molecular basis of high‐altitude adaptation in deer mice. PLoS Genet. 3:e45. 17397259 10.1371/journal.pgen.0030045 PMC1839143

[evo13150-bib-0063] Tam, P. P. 1981 The control of somitogenesis in mouse embryos. J. Embryol. Exp. Morphol. 65(Suppl):103–128. 6801176

[evo13150-bib-0064] Taylor, Z. S. , and S. M. Hoffman . 2012 Microsatellite genetic structure and cytonuclear discordance in naturally fragmented populations of deer mice (*Peromyscus maniculatus*). J. Hered. 103:71–79. 21976772 10.1093/jhered/esr100

[evo13150-bib-0065] Theiler, K. 1989 The house mouse—Atlas of embryonic development. Springer‐Verlag, New York, NY.

[evo13150-bib-0066] Thompson, D. B. 1990 Different spatial scales of adaptation in the climbing behavior of *Peromyscus maniculatus*: geographic variation, natural selection, and gene flow. Evolution 44:952–965. 10.1111/j.1558-5646.1990.tb03817.x 28569032

[evo13150-bib-0067] Van der Auwera, G. A. , M. O. Carneiro , C. Hartl , R. Poplin , G. del Angel , A. Levy‐Moonshine , T. Jordan , K. Shakir , D. Roazen , J. Thibault , et al. 2013 From FastQ data to high‐confidence variant calls: the genome analysis toolkit best practices pipeline. Curr. Protoc. Bioinformatics 43:11.10.1–11.10.33. 25431634 10.1002/0471250953.bi1110s43 PMC4243306

[evo13150-bib-0068] Weir, B. S. , and C. C. Cockerham . 1984 Estimating F‐statistics for the analysis of population structure. Evolution 38:1358–1370. 10.1111/j.1558-5646.1984.tb05657.x 28563791

[evo13150-bib-0069] Yang, D.‐S. , and G. J. Kenagy . 2009 Nuclear and mitochondrial DNA reveal contrasting evolutionary processes in populations of deer mice (*Peromyscus maniculatus*). Mol. Ecol. 18:5115–5125. 19912541 10.1111/j.1365-294X.2009.04399.x

[evo13150-bib-0070] Yang, D.‐S. , and G. J. Kenagy . 2011 Population delimitation across contrasting evolutionary clines in deer mice (*Peromyscus maniculatus*). Ecol. Evol. 1:26–36. 22393480 10.1002/ece3.3 PMC3287378

[evo13150-bib-0071] Zheng, X. , B. S. Arbogast , and G. J. Kenagy . 2003 Historical demography and genetic structure of sister species: deermice (*Peromyscus*) in the North American temperate rain forest. Mol. Ecol. 12:711–724. 12675826 10.1046/j.1365-294x.2003.01770.x

