# Supplementary material for: The ultimate and proximate mechanisms driving the evolution of long tails in forest deer mice
Source: Evolution. 2016 Dec 27;71(2):261–73. doi: 10.1111/evo.13150 (PMC5324611; doi:10.1111/evo.13150)
Supplement: Supplementary file 1 — Figure S1. Neighbor‐joining trees based on Euclidean distances in principal component space. Figure S2. Maximum Likelihood phylogeny of P. maniculatus individuals based on >14000 nuclear SNPs. Figure S3. Maximum Likelihood phylogeny of P. maniculatus individuals based on the mitochondrial CO3‐ND3 fragment. Figure S4. Pairwise weighted FST among population samples containing >3 individuals. [file EVO-71-261-s001.docx]

**The ultimate and proximate mechanisms driving the evolution of long tails in forest deer mice**

**Supplemental Figures**

Evan P. Kingsley^1^*, Krzysztof M. Kozak^2†^*, Susanne P. Pfeifer^3^, Dou-Shuan Yang^4‡^, and Hopi E. Hoekstra^1^

*^1^Howard Hughes Medical Institute, Department of Organismic and Evolutionary Biology, Department of Molecular and Cellular Biology, Museum of Comparative Zoology, Harvard University, 16 Divinity Avenue, Cambridge, Massachusetts 02138, USA*

*^2^Department of Zoology, University of Cambridge, Cambridge CB2 3EJ, UK*

*^3^School of Life Sciences, P.O. Box 874501, Arizona State University,* *Tempe, AZ 85287, USA*

*^4^Burke Museum and Department of Biology, Box 351800, University of Washington, Seattle, Washington 98195, USA*

*These authors contributed equally to this work.

^†^ Current address: Smithsonian Tropical Research Institute, Apartado Postal 0843-03092, Panamá, República de Panamá

^‡^Current address: US Fish and Wildlife Service, Ventura Field Office, 2493 Portola Road #B, Ventura, California 93003, USA

**Supplemental Figure 1. Neighbor-joining trees based on Euclidean distances in principal component space.** Cladogram based on Euclidian distances in PC1 and PC2 allows visualization of genetic clustering.

**Supplemental Figure 2. Maximum Likelihood phylogeny of *P. maniculatus* individuals based on >14000 nuclear SNPs.** Branch lengths estimated under the GTR+GAMMA model with an ascertainment bias correction. Bootstrap support values >50 reported at the nodes. Notice at least two separate clades with forest ecotypes. Colors indicate local GIS land cover-defined habitat (tan = prairie, green = forest).

**Supplemental Figure 3. Maximum Likelihood phylogeny of *P. maniculatus* individuals based on the mitochondrial CO3-ND3 fragment.** Bootstrap support values >50 indicated where relevant. NB: this tree is based on a different choice of populations than the PCA and ML analyses. Colors indicate habitat based on subspecies description (tan = prairie, green = forest, grey = mixed).

**Supplemental Figure 4. Pairwise weighted F_ST_ among population samples containing >3 individuals.** We estimated F_ST_ with the method of Weir and Cockerham (1984) as implemented in VCFtools (Danecek *et al.* 2011).
