# Supplementary material for: The ultimate and proximate mechanisms driving the evolution of long tails in forest deer mice
Source: Evolution. 2016 Dec 27;71(2):261–73. doi: 10.1111/evo.13150 (PMC5324611; doi:10.1111/evo.13150)
Supplement: Supplementary file 2 — Table S1. Samples genotyped in the genome‐wide capture. [file EVO-71-261-s002.docx]

| **ID** | **state** | **lat** | **long** | **subspp** | **forest/prairie** | **in population contrasts** | **in individual contrasts** | **x-ray samples** | **source** | **accession** |
| --- | --- | --- | --- | --- | --- | --- | --- | --- | --- | --- |
| abietorum_ME_01 | ME | 45.998 | -68.907 | *abietorum* | 1 |  | x |  | SI | SI569775 |
| bairdii_NE_01 | NE | 42.190 | -98.040 | *bairdii* | 0 |  | x |  | TTU | TTU75979 |
| bairdii_NE_02 | NE | 42.190 | -98.040 | *bairdii* | 0 |  | x |  | TTU | TTU75938 |
| blandus_CH_01 | Chihuahua | 27.037 | -105.244 | *blandus* | 0 |  | x |  | NMMNH | NMMNH5380 |
| blandus_CH_02 | Chihuahua | 27.037 | -105.244 | *blandus* | 0 |  | x |  | NMMNH | NMMNH5381 |
| blandus_NM_01 | NM | 33.075 | -106.003 | *blandus* | 0 | blandus_NM | x |  | RM/MCZ |  |
| blandus_NM_02 | NM | 33.075 | -106.003 | *blandus* | 0 | blandus_NM | x |  | RM/MCZ |  |
| blandus_NM_03 | NM | 33.075 | -106.003 | *blandus* | 0 | blandus_NM | x |  | RM/MCZ |  |
| blandus_NM_04 | NM | 33.075 | -106.003 | *blandus* | 0 | blandus_NM | x |  | RM/MCZ |  |
| blandus_NM_05 | NM | 33.075 | -106.003 | *blandus* | 0 | blandus_NM | x |  | RM/MCZ |  |
| blandus_NM_06 | NM | 33.075 | -106.003 | *blandus* | 0 | blandus_NM | x |  | RM/MCZ |  |
| borealis_AB_01 | Alberta | 50.733 | -113.983 | *borealis* | 0 |  | x |  | ROM | ROM109759 |
| borealis_AB_02 | Alberta | 53.406 | -113.541 | *borealis* | 0 |  | x |  | TTU | TTU55496 |
| borealis_AB_03 | Alberta | 51.997 | -114.043 | *borealis* | 0 |  | x |  | TTU | TTU71347 |
| borealis_AB_04 | Alberta | 50.182 | -111.236 | *borealis* | 0 |  | x |  | TTU | TTU71362 |
| fulvus_TL_01 | Tlaxcala | 19.281 | -98.367 | *fulvus* | 0 |  | x |  | TTU | TTU82708 |
| fulvus_VZ_01 | Veracruz | 19.570 | -97.250 | *fulvus* | 0 |  | x |  | TTU | TTU104953 |
| gambelii_CA_01 | CA | 32.945 | -117.168 | *gambelii* | 0 |  | x |  | SI | SI569175 |
| gambelii_CA_02 | CA | 37.741 | -119.408 | *gambelii* | 0 |  | x |  | MVZ | MVZ208143 |
| gambelii_CA_03 | CA | 34.415 | -119.880 | *gambelii* | 0 |  | x |  | MVZ | MVZ216082 |
| gambelii_CA_04 | CA | 34.415 | -119.880 | *gambelii* | 0 |  | x |  | MVZ | MVZ216101 |
| gambelii_CA_05 | CA | 32.945 | -117.168 | *gambelii* | 0 |  | x |  | SI | SI569236 |
| gambelii_CA_06 | CA | 33.125 | -116.675 | *gambelii* | 0 |  | x |  | SI | SI569270 |
| gambelii_CA_07 | CA | 33.508 | -116.675 | *gambelii* | 0 |  | x |  | SI | SI569278 |
| gambelii_CA_08 | CA | 32.552 | -117.000 | *gambelii* | 0 |  | x |  | SI | SI569292 |
| gambelii_CA_09 | CA | 37.740 | -119.396 | *gambelii* | 1 |  | x |  | MVZ | MVZ208126 |
| gambelii_OR_01 | OR | 42.184 | -117.502 | *gambelii* | 0 | gambelii_OR | x | x | DSY |  |
| gambelii_OR_02 | OR | 42.184 | -117.502 | *gambelii* | 0 | gambelii_OR | x | x | DSY |  |
| gambelii_OR_03 | OR | 42.184 | -117.502 | *gambelii* | 0 | gambelii_OR | x | x | DSY |  |
| gambelii_OR_04 | OR | 42.184 | -117.502 | *gambelii* | 0 | gambelii_OR | x | x | DSY |  |
| gambelii_OR_05 | OR | 42.184 | -117.502 | *gambelii* | 0 | gambelii_OR | x | x | DSY |  |
| gambelii_OR_06 | OR | 42.184 | -117.502 | *gambelii* | 0 | gambelii_OR | x | x | DSY |  |
| gambelii_OR_07 | OR | 42.184 | -117.502 | *gambelii* | 0 | gambelii_OR | x | x | DSY |  |
| gambelii_OR_08 | OR | 42.184 | -117.502 | *gambelii* | 0 | gambelii_OR | x | x | DSY |  |
| gambelii_OR_09 | OR | 42.184 | -117.502 | *gambelii* | 0 | gambelii_OR | x | x | DSY |  |
| gracilis_MI_01 | MI | 46.020 | -84.430 | *gracilis* | 1 | gracilis_MI | x |  | MCZ |  |
| gracilis_MI_02 | MI | 46.020 | -84.430 | *gracilis* | 1 | gracilis_MI | x |  | MCZ |  |
| gracilis_MI_03 | MI | 46.020 | -84.430 | *gracilis* | 1 | gracilis_MI | x |  | MCZ |  |
| gracilis_MI_04 | MI | 46.020 | -84.430 | *gracilis* | 1 | gracilis_MI | x |  | MCZ |  |
| gracilis_MI_05 | MI | 46.020 | -84.430 | *gracilis* | 1 | gracilis_MI | x |  | MCZ |  |
| gracilis_MI_06 | MI | 46.020 | -84.430 | *gracilis* | 1 | gracilis_MI | x |  | MCZ |  |
| gracilis_MI_07 | MI | 46.020 | -84.430 | *gracilis* | 1 | gracilis_MI | x |  | MCZ |  |
| gracilis_ON_01 | Ontario | 45.000 | -78.500 | *gracilis* | 1 | gracilis_ON | x |  | ROM | ROM98940 |
| gracilis_ON_02 | Ontario | 45.000 | -78.500 | *gracilis* | 1 | gracilis_ON | x |  | ROM | ROM97094 |
| gracilis_ON_03 | Ontario | 45.000 | -78.500 | *gracilis* | 1 | gracilis_ON | x |  | ROM | ROM97095 |
| keeni_AK_01 | AK | 56.230 | -132.970 | *keeni* | 1 |  |  |  | UAM | UAF20877 |
| keeni_AK_02 | AK | 56.230 | -132.970 | *keeni* | 1 |  |  |  | UAM | UAF20880 |
| nebrascensis_UT_01 | UT | 38.819 | -109.773 | *nebrascensis* | 0 |  | x |  | MVZ | MVZ199465 |
| nebrascensis_UT_02 | UT | 38.819 | -109.773 | *nebrascensis* | 0 |  | x |  | MVZ | MVZ199466 |
| nebrascensis_WY_01 | WY | 40.967 | -109.604 | *nebrascensis* | 0 |  | x |  | TTU | TTU42218 |
| nubiterrae_PA_01 | PA | 40.145 | -79.268 | *nubiterrae* | 1 | nubiterrae_PA | x | x | ARY/MCZ |  |
| nubiterrae_PA_02 | PA | 40.145 | -79.268 | *nubiterrae* | 1 | nubiterrae_PA | x | x | ARY/MCZ |  |
| nubiterrae_PA_03 | PA | 40.145 | -79.268 | *nubiterrae* | 1 | nubiterrae_PA | x | x | ARY/MCZ |  |
| nubiterrae_PA_04 | PA | 40.145 | -79.268 | *nubiterrae* | 1 | nubiterrae_PA | x | x | ARY/MCZ |  |
| nubiterrae_PA_05 | PA | 40.145 | -79.268 | *nubiterrae* | 1 | nubiterrae_PA | x | x | ARY/MCZ |  |
| nubiterrae_PA_06 | PA | 40.145 | -79.268 | *nubiterrae* | 1 | nubiterrae_PA | x | x | ARY/MCZ |  |
| nubiterrae_PA_07 | PA | 40.145 | -79.268 | *nubiterrae* | 1 | nubiterrae_PA | x | x | ARY/MCZ |  |
| nubiterrae_VA_01 | VA | 38.414 | -79.580 | *nubiterrae* | 1 |  | x |  | SI | SI570138 |
| rubidus_CA_01 | CA | 37.994 | -122.501 | *rubidus* | 1 |  | x |  | MVZ | MVZ219039 |
| rubidus_CA_02 | CA | 37.994 | -122.501 | *rubidus* | 1 |  | x |  | MVZ | MVZ219041 |
| rubidus_OR_01 | OR | 42.345 | -124.206 | *rubidus* | 1 | rubidus_OR | x | x | DSY |  |
| rubidus_OR_02 | OR | 42.345 | -124.206 | *rubidus* | 1 | rubidus_OR | x | x | DSY |  |
| rubidus_OR_03 | OR | 42.345 | -124.206 | *rubidus* | 1 | rubidus_OR | x | x | DSY |  |
| rubidus_OR_04 | OR | 42.345 | -124.206 | *rubidus* | 1 | rubidus_OR | x | x | DSY |  |
| rubidus_OR_05 | OR | 42.345 | -124.206 | *rubidus* | 1 | rubidus_OR | x | x | DSY |  |
| rubidus_OR_06 | OR | 42.345 | -124.206 | *rubidus* | 1 | rubidus_OR | x | x | DSY |  |
| rubidus_OR_07 | OR | 42.345 | -124.206 | *rubidus* | 1 | rubidus_OR | x | x | DSY |  |
| rubidus_OR_08 | OR | 42.345 | -124.206 | *rubidus* | 1 | rubidus_OR | x | x | DSY |  |
| rubidus_OR_09 | OR | 42.345 | -124.206 | *rubidus* | 1 | rubidus_OR | x | x | DSY |  |
| rufinus_NM_01 | NM | 35.240 | -108.768 | *rufinus* | 0 |  |  |  | MSB | MSB98501 |
| rufinus_NM_02 | NM | 35.240 | -106.768 | *rufinus* | 0 |  |  |  | MSB | MSB96295 |
| sonoriensis_AZ_01 | AZ | 35.080 | -109.800 | *sonoriensis* | 0 |  |  |  | MSB | MSB122974 |
| sonoriensis_AZ_02 | AZ | 35.340 | -111.670 | *sonoriensis* | 1 |  |  |  | LT/MCZ |  |
| sonoriensis_AZ_03 | AZ | 35.080 | -109.800 | *sonoriensis* | 0 |  |  |  | MSB | MSB122975 |
| sonoriensis_CA_01 | CA | 37.910 | -119.110 | *sonoriensis* | 0 |  |  |  | LT/MCZ |  |
| sonoriensis_CA_012 | CA | 37.910 | -119.110 | *sonoriensis* | 0 |  |  |  | LT/MCZ |  |
| not genotyped | MI | Washtenaw Co |  | *bairdii* | 0 |  |  | x | UMMZ | UMMZ52800 |
| not genotyped | MI | Washtenaw Co |  | *bairdii* | 0 |  |  | x | UMMZ | UMMZ52801 |
| not genotyped | MI | Washtenaw Co |  | *bairdii* | 0 |  |  | x | UMMZ | UMMZ55422 |
| not genotyped | MI | Washtenaw Co |  | *bairdii* | 0 |  |  | x | UMMZ | UMMZ55428 |
| not genotyped | MI | Washtenaw Co |  | *bairdii* | 0 |  |  | x | UMMZ | UMMZ55429 |
| not genotyped | MI | Washtenaw Co |  | *bairdii* | 0 |  |  | x | UMMZ | UMMZ116396 |
| not genotyped | MI | Washtenaw Co |  | *bairdii* | 0 |  |  | x | UMMZ | UMMZ116397 |
| not genotyped | MI | Washtenaw Co |  | *bairdii* | 0 |  |  | x | UMMZ | UMMZ116398 |
| not genotyped | MI | Washtenaw Co |  | *bairdii* | 0 |  |  | x | UMMZ | UMMZ126330 |
| not genotyped | MI | Washtenaw Co |  | *bairdii* | 0 |  |  | x | UMMZ | UMMZ163792 |
| not genotyped | MI | Washtenaw Co |  | *bairdii* | 0 |  |  | x | UMMZ | UMMZ93504 |
| not genotyped | MI | Washtenaw Co |  | *bairdii* | 0 |  |  | x | UMMZ | UMMZ93505 |
| not genotyped | PA | 40.145 | -79.268 | *nubiterrae* | 1 |  |  | x | EPK |  |
| not genotyped | PA | 40.145 | -79.268 | *nubiterrae* | 1 |  |  | x | EPK |  |
| not genotyped | PA | 40.145 | -79.268 | *nubiterrae* | 1 |  |  | x | EPK |  |
| not genotyped | PA | 40.145 | -79.268 | *nubiterrae* | 1 |  |  | x | EPK |  |
| not genotyped | PA | 40.145 | -79.268 | *nubiterrae* | 1 |  |  | x | EPK |  |
| ARY-Pm-3 |  |  |  | *P. leucopus* |  |  |  |  | ARY/MCZ |  |
| EPK27 |  |  |  | *P. leucopus* |  |  |  |  | MCZ |  |
| RM2 |  |  |  | *P. leucopus* |  |  |  |  | RM/MCZ |  |
| RM3 |  |  |  | *P. leucopus* |  |  |  |  | RM/MCZ |  |
|  |  |  |  |  |  |  |  |  |  |  |
| **Supplemental Table 1. Samples genotyped in the genome-wide capture.** Habitat classified as grassland/prairie (0) or forest (1) based on GIS. Samples collected by individuals: RM – Ricardo Mallarino; LT – Leslie Turner; ARY – Adrian R. Young; DSY – Dou-Shuan Yang; EPK – Evan P. Kingsley. Samples from institutions: SI - Smithsonian Institution (DC); MSB – Museum of Southwestern Biology; MVZ – Museum of Vertebrate Zoology; ROM – Royal Ontario Museum; UMNH – Utah Museum of Natural History; NMMNH – New Mexico Museum of Nat. Hist.; TTU – Texas Tech University; UAM – University of Alaska Museum; UMMZ – University of Michigan Museum of Zoology. | | | | | | | | | | |
