# Supplementary material for: The ultimate and proximate mechanisms driving the evolution of long tails in forest deer mice
Source: Evolution. 2016 Dec 27;71(2):261–73. doi: 10.1111/evo.13150 (PMC5324611; doi:10.1111/evo.13150)
Supplement: Supplementary file 3 — Table S2. Samples genotyped at the mitochondrion. [file EVO-71-261-s003.docx]

| **#** | **Subspecies** | **State** | **Location** | **Lat** | **Long** | **Collection** | **ID** | **Capture** |
| --- | --- | --- | --- | --- | --- | --- | --- | --- |
| 1 | *abietorum* | ME | Somerset | 45.998 | -68.907 | SI | 569775 | Yes |
| 2 | *gambelii* | CA | San Diego | 32.945 | -117.168 | SI | 569175 | Yes |
| 3 | *gambelii* | CA | San Diego | 32.945 | -117.168 | SI | 569236 | Yes |
| 4 | *gambelii* | CA | San Diego | 33.125 | -116.675 | SI | 569270 | Yes |
| 5 | *gambelii* | CA | San Diego | 33.508 | -116.675 | SI | 569278 | Yes |
| 6 | *gambelii* | CA | San Diego | 32.552 | -117.000 | SI | 569292 | Yes |
| 7 | *gracilis* | VT | Orleans | 44.750 | -72.051 | SI | 568236 |  |
| 9 | *nubiterrae* | VA | Highland | 38.414 | -79.580 | SI | 570138 | Yes |
| 10 | *rufinus* | CO | Whites Canyon | 37.228 | -108.374 | MSB | 121533 |  |
| 11 | *nebrascensis* | MT | Hyalite | 45.554 | -111.040 | MSB | 56716 |  |
| 12 | *austerus* | WA | Fort Lewis | 47.090 | -122.604 | MSB | 86381 |  |
| 13 | *rufinus* | NM | Navajo | 35.240 | -108.768 | MSB | 98501 | Yes |
| 14 | *rufinus* | NM | Navajo | 35.240 | -106.768 | MSB | 96295 | Yes |
| 15 | *rufinus* | NM | Placitas | 35.303 | -106.484 | MSB | 150561 |  |
| 16 | *rufinus* | CO | Mesa Verde NP | 37.228 | -108.374 | MSB | 121512 |  |
| 17 | *sonoriensis* | UT | Bromide Basin | 38.060 | -110.790 | MSB | 122773 |  |
| 18 | *sonoriensis* | UT | Bromide Basin | 38.060 | -110.790 | MSB | 122777 |  |
| 19 | *sonoriensis* | AZ | Petrified Forest NP | 35.080 | -109.800 | MSB | 122974 | Yes |
| 20 | *sonoriensis* | AZ | Petrified Forest NP | 35.080 | -109.800 | MSB | 122975 |  |
| 21 | *sonoriensis* | NM | Placitas | 35.303 | -106.484 | MSB | 10002615 |  |
| 23 | *coolidgei* | BS | El Rosario | 30.108 | -115.760 | MVZ | 159779 |  |
| 24 | *sonoriensis* | CA | Metcalf Meadows | 34.224 | -116.938 | MVZ | 198753 |  |
| 25 | *sonoriensis* | CA | Metcalf Meadows | 34.225 | -116.940 | MVZ | 198756 |  |
| 26 | *rubidus* | CA | Angelo Coast Res. | 39.741 | -123.631 | MVZ | 199143 |  |
| 27 | *nebrascensis* | UT | Rock Canyon Corral | 38.819 | -109.773 | MVZ | 199465 | Yes |
| 28 | *nebrascensis* | UT | Rock Canyon Corral | 38.819 | -109.773 | MVZ | 199466 | Yes |
| 29 | *gambelii* | CA | Yosemite | 37.740 | -119.396 | MVZ | 208126 | Yes |
| 30 | *gambelii* | CA | Yosemite | 37.741 | -119.408 | MVZ | 208143 | Yes |
| 31 | *gambelii* | CA | UC Santa Barbara | 34.415 | -119.880 | MVZ | 216082 | Yes |
| 32 | *gambelii* | CA | UC Santa Barbara | 34.415 | -119.880 | MVZ | 216101 | Yes |
| 33 | *sonoriensis* | CA | Mazourka Canyon | 36.957 | -118.093 | MVZ | 216544 |  |
| 34 | *rubidus* | OR | Curry | 42.818 | -124.481 | MVZ | 216767 |  |
| 35 | *santacruzae* | CA | Santa Cruz Island | 33.996 | -119.728 | MVZ | 216957 |  |
| 36 | *santacruzae* | CA | Santa Cruz Island | 33.996 | -119.728 | MVZ | 216959 |  |
| 37 | *santacruzae* | CA | Santa Cruz Island | 33.996 | -119.728 | MVZ | 216960 |  |
| 38 | *rubidus* | CA | Six Rivers NF | 40.891 | -123.583 | MVZ | 217522 |  |
| 39 | *rubidus* | CA | Six Rivers NF | 40.891 | -123.583 | MVZ | 217538 |  |
| 40 | *rubidus* | CA | China Camp SP | 37.994 | -122.501 | MVZ | 219039 | Yes |
| 41 | *rubidus* | CA | China Camp SP | 37.994 | -122.501 | MVZ | 219041 | Yes |
| 42 | *rubidus* | OR | Curry | 42.818 | -124.481 | MVZ | 219638 |  |
| 43 | *gracilis* | ON | Cochrane | 45.000 | -78.500 | ROM | 98940 | Yes |
| 44 | *gracilis* | ON | Cochrane | 45.000 | -78.500 | ROM | 98941 |  |
| 45 | *austerus/oreas* | BC | Maple Ridge | 49.267 | -122.500 | ROM | 109746 |  |
| 46 | *austerus/oreas* | BC | Maple Ridge | 49.267 | -122.500 | ROM | 109747 |  |
| 47 | *borealis* | AB | Okotoks | 50.733 | -113.983 | ROM | 109759 | Yes |
| 48 | *borealis* | AB | Okotoks | 50.733 | -113.983 | ROM | 109767 |  |
| 50 | *abietorum* | NS | Cole Harbour | 44.649 | -63.4831 | ROM | 110289 |  |
| 51 | *gracilis* | ON | Long Point | 43.950 | -78.950 | ROM | 112766 |  |
| 52 | *gracilis* | ON | Long Point | 43.950 | -78.950 | ROM | 112767 |  |
| 53 | *P. leucopus* | ON | Durham | 43.950 | -78.950 | ROM | 112739 |  |
| 55 | *gracilis* | ON | Haliburton | 45.000 | -78.500 | ROM | 97094 | Yes |
| 56 | *gracilis* | ON | Haliburton | 45.000 | -78.500 | ROM | 97095 | Yes |
| 57 | *gambelii* | OR | Alkali Lake | 42.247 | -121.485 | ROM | 12512 |  |
| 58 | *gambelii* | OR | Alkali Lake | 42.247 | -121.485 | ROM | 112513 |  |
| 59 | *rufinus* | UT | Summit | 40.600 | -111.017 | UMNH | 30557 |  |
| 60 | *rufinus* | UT | Summit | 40.600 | -111.017 | UMNH | 30558 |  |
| 61 | *sonoriensis* | UT | Juab | 39.927 | -112.172 | UMNH | 31943 |  |
| 62 | *sonoriensis* | UT | Juab | 39.925 | -112.171 | UMNH | 31944 |  |
| 63 | *sonoriensis* | NV | Elko | 40.956 | -115.527 | UMNH | 32368 |  |
| 64 | *sonoriensis* | NV | Elko | 40.956 | -115.527 | UMNH | 32369 |  |
| 66 | *serratus* | ID | Elmore | 43.800 | -115.120 | NMMNH | 3758 |  |
| 67 | *serratus* | ID | Elmore | 43.800 | -115.120 | NMMNH | 3769 |  |
| 68 | *rufinus* | NM | Coyote Creek | 36.239 | -105.239 | NMMNH | 4041 |  |
| 69 | *blandus* | CH | Jimenez | 27.037 | -105.244 | NMMNH | 5380 | Yes |
| 70 | *blandus* | CH | Jimenez | 27.037 | -105.244 | NMMNH | 5381 | Yes |
| 72 | *borealis* | AB | Edmonton | 53.406 | -113.541 | TTU | 55496 | Yes |
| 73 | *borealis* | AB | Red Deer | 51.997 | -114.043 | TTU | 71347 | Yes |
| 74 | *borealis* | AB | Cypress | 50.182 | -111.236 | TTU | 71362 | Yes |
| 75 | *serratus* | ID | Lola Creek | 44.400 | -115.207 | TTU | 55614 |  |
| 76 | *bairdii* | NE | Antelope | 42.190 | -98.040 | TTU | 75979 | Yes |
| 77 | *bairdii* | NE | Antelope | 42.190 | -98.040 | TTU | 75938 | Yes |
| 79 | *nebrascensis* | WY | Sweetwater | 40.967 | -109.604 | TTU | 42218 | Yes |
| 83 | *bairdii* | AR | Mississippi | 35.820 | -90.070 | TTU | 97830 |  |
| 84 | *labecula* | TL | Tepetitla | 19.281 | -98.367 | TTU | 82708 |  |
| 86 | *fulvus* | VZ | Perote | 19.570 | -97.250 | TTU | 104953 | Yes |
| 87 | *angustus* | BC | Vancouver Island | 48.990 | -123.810 | TTU | 71416 |  |
| 88 | *angustus* | BC | Vancouver Island | 48.990 | -123.810 | TTU | 71418 |  |
| 90 | *austerus* | WA | Pierce | 47.110 | -122.560 | TTU | 100410 |  |
| 91 | *austerus* | WA | Pierce | 47.110 | -122.560 | TTU | 100411 |  |
| 92 | *nubiterrae* | PA | Laurel Summit | 40.145 | -79.268 | HL | ARY PM 1 | Yes |
| 93 | *nubiterrae* | PA | Laurel Summit | 40.146 | -79.269 | HL | ARY PM 5 | Yes |
| 94 | *rufinus* | AZ | Humphreys Peak | 35.340 | -111.670 | HL | LMT 120 |  |
| 95 | *rufinus* | AZ | Humphreys Peak | 35.340 | -111.670 | HL | LMT 121 |  |
| 96 | *sonoriensis* | CA | William's Butte | 37.910 | -119.110 | HL | LMT 264 |  |
| 97 | *sonoriensis* | CA | William's Butte | 37.910 | -119.110 | HL | LMT 272 |  |
| 98 | *luteus* | CO | Clear Creek | 39.690 | -105.640 | HL | JFS 80 |  |
| 99 | *luteus* | CO | Clear Creek | 39.690 | -105.640 | HL | JFS 82 |  |
| 100 | *luteus* | CO | Yuma | 40.120 | -102.710 | HL | JFS 114 |  |
| 101 | *luteus* | CO | Yuma | 40.120 | -102.710 | HL | JFS 117 |  |
| 102 | *luteus* | NE | Cherry | 42.878 | -100.260 | HL | CRL 34 |  |
| 103 | *luteus* | NE | Cherry | 42.446 | -100.640 | HL | CRL 183 |  |
| 104 | *keeni* | AK | Shrubby Island | 56.233 | -132.967 | UAM | AK 20877 | Yes |
| 105 | *keeni* | AK | Shrubby Island | 56.233 | -132.967 | UAM | AK 20880 | Yes |
| 106 | *P. polionotus* | GA | Statesboro | 32.440 | -81.760 | HL | O3.041 |  |
| 108 | *gracilis* | MI | Hiawatha NP | 46.048 | -84.732 | UMMZ | 172494 |  |
| 109 | *gracilis* | MI | Hiawatha NP | 47.048 | -83.732 | UMMZ | 166945 |  |
| 111 | *gracilis* | MI | Hiawatha NP | 46.020 | -84.430 | HL | EPK 003 |  |
| 112 | *gracilis* | MI | Hiawatha NP | 46.020 | -84.430 | HL | EPK 004 |  |

**Supplementary Table 2. Samples genotyped at the mitochondrion.** Samples included in the capture indicated. Missing numbers are samples removed due to contamination/mislabeling. SI: Smithsonian Institution (DC); MSB: Museum of Southwestern Biology; MVZ: Museum of Vertebrate Zoology; ROM: Royal Ontario Museum; UMNH: Utah Museum of Natural History; NMMNH: New Mexico Museum of Nat. Hist.; TTU: Texas Tech University; UAM: University of Alaska Museum; UMMZ: University of Michigan Museum of Zoology; HL: Hoekstra Lab.
